# COVID-19-related strokes are associated with increased mortality and morbidity: A multicenter comparative study from Bengaluru, South India

**DOI:** 10.1177/1747493020968236

**Published:** 2020-12-06

**Authors:** Thomas Mathew, Saji K John, GRK Sarma, Raghunandan Nadig, Shiva Kumar R, Uday Murgod, Manjunath Mahadevappa, Mahendra Javali, Purushottam Thammaya Acharya, Guruprasad Hosurkar, Pramod Krishnan, Vikram Kamath, Sagar Badachi, Delon D Souza, Rajesh B Iyer, Rajesh Karalumangala Nagarajaiah, Bawani Anand, Sujit Kumar, Suresh Kodapala, Sonia Shivde, Amrutha Avati, Rohit Baddala, Prasanth Babu Potharlanka, Sravanthi Pavuluri, Abhinaya Varidireddy, Poonam Awatare, Nandavar Shobha, Umashankar Renukaradhya, S Praveen Kumar, Jayachandran Ramachandran, Ramesh Arumugam, Saikanth Deepalam, Sharath Kumar, Vikram Huded

**Affiliations:** 1 Department of Neurology, St. John’s Medical College Hospital, Bengaluru, Karnataka, India; 2 Department of Neurology, 477861 Sakra World Hospital, Bengaluru, Karnataka, India; 3 Department of Neurology, Manipal Hospitals, Bengaluru, Karnataka, India; 4 Department of Neurology, Ramaiah Memorial Hospital, Bengaluru, Karnataka, India; 5 Department of Neurology, Columbia Asia Hospital 6/4, Bengaluru, Karnataka, India; 6 Department of Neurology, Apollo Hospital, Bannerghatta, Bengaluru, Karnataka, India; 7 Department of Neurology, Vikram Hospital, Anne’s College, Bengaluru, Karnataka, India; 8 Department of Neurology, People Tree Hospitals, Bengaluru, Karnataka, India; 9 Department of Neurology, Apollo Hospitals, Sheshadripuram, Bengaluru, Karnataka, India; 10 Department of Neurology, Vydehi Institute of Medical Science, Bangalore, India; 11 Department of Neurology, Bangalore Neuro Centre, Bengaluru, Karnataka, India; 12 Department of Neurology, 29099 Bangalore Medical College and Research Institute, Bengaluru, Karnataka, India; 13 Department of Neurology, NH Institute of Neurosciences, Mazumdar Shaw Medical Center, Bengaluru, Karnataka, India

**Keywords:** COVID-19, SARS–CoV2, ischemic stroke, thrombolysis, mortality, morbidity, India

## Abstract

**Background:**

COVID-19-related strokes are increasingly being diagnosed across the world. Knowledge about the clinical profile, imaging findings, and outcomes is still evolving. Here we describe the characteristics of a cohort of 62 COVID-19-related stroke patients from 13 hospitals, from Bangalore city, south India.

**Objective:**

To describe the clinical profile, neuroimaging findings, interventions, and outcomes in COVID-19-related stroke patients.

**Methods:**

This is a multicenter retrospective study of all COVID-19-related stroke patients from 13 hospitals from south India; 1st June 2020–31st August 2020. The demographic, clinical, laboratory, and neuroimaging data were collected along with treatment administered and outcomes. SARS–CoV-2 infection was confirmed in all cases by RT-PCR testing. The data obtained from the case records were entered in SPSS 25 for statistical analysis.

**Results:**

During the three-month period, we had 62 COVID-19-related stroke patients, across 13 centers; 60 (97%) had ischemic strokes, while 2 (3%) had hemorrhagic strokes. The mean age of patients was 55.66 ± 13.20 years, with 34 (77.4%) males. Twenty-six percent (16/62) of patients did not have any conventional risk factors for stroke. Diabetes mellitus was seen in 54.8%, hypertension was present in 61.3%, coronary artery disease in 8%, and atrial fibrillation in 4.8%. Baseline National Institutes of Health Stroke Scale score was 12.7 ± 6.44. Stroke severity was moderate (National Institutes of Health Stroke Scale 5–15) in 27 (61.3%) patients, moderate to severe (National Institutes of Health Stroke Scale 16–20) in 13 (20.9%) patients and severe (National Institutes of Health Stroke Scale 21–42) in 11 (17.7%) patients. According to TOAST classification, 48.3% was stroke of undetermined etiology, 36.6% had large artery atherosclerosis, 10% had small vessel occlusion, and 5% had cardioembolic strokes. Three (5%) received intravenous thrombolysis with tenecteplase 0.2 mg/kg and 3 (5%) underwent mechanical thrombectomy, two endovascular and one surgical. Duration of hospital stay was 16.16 ± 6.39 days; 21% (13/62) died in hospital, while 37 (59.7%) had a modified Rankin score of 3–5 at discharge. Hypertension, atrial fibrillation, and higher baseline National Institutes of Health Stroke Scale scores were associated with increased mortality. A comparison to 111 historical controls during the non-COVID period showed a higher proportion of strokes of undetermined etiology, higher mortality, and higher morbidity in COVID-19-related stroke patients.

**Conclusion:**

COVID-19-related strokes are increasingly being recognized in developing countries, like India. Stroke of undetermined etiology appears to be the most common TOAST subtype of COVID-19-related strokes. COVID-19-related strokes were more severe in nature and resulted in higher mortality and morbidity. Hypertension, atrial fibrillation, and higher baseline National Institutes of Health Stroke Scale scores were associated with increased mortality.

## Introduction

Recent evidence surfacing across the globe suggests that SARS–CoV-2 infection is associated with both ischemic and hemorrhagic strokes. Stroke appears to be one of the dangerous neurological complications of SARS–CoV-2 infection. Of late, with surges in COVID-19 cases in India, especially in the state of Karnataka, we are witnessing an increasing number of COVID-19-related strokes. The knowledge about the clinical profile, imaging findings, and outcomes of COVID-19-related strokes is still evolving. Here we have described the characteristics of COVID-19-related strokes from 13 stroke centers from Bengaluru, an urban city in the state of Karnataka, south India. We have compared these observations with a cohort of non-COVID strokes, during the same months of the previous year and analyzed the factors associated with mortality in COVID-19-related stroke patients.

## Methods

This multicenter retrospective observational study was conducted in 13 stroke treatment ready hospitals in an urban city (Bengaluru) in south India from 1st June 2020 to 31st August 2020. All consecutive cases of COVID-related strokes from 13 centers were recruited during the study period. The presence of stroke was confirmed in all cases either by CT (computed tomography) or MRI (magnetic resonance imaging) of the brain. All patients underwent RT-PCR testing on the nasopharyngeal swab. Patients with strokes and positive RT-PCR for SARS–CoV 2 were enrolled for the study. Data regarding demographic variables, comorbidities, clinical features, National Institutes of Health Stroke Scale (NIHSS) and modified Rankin score (mRS) at baseline and discharge, laboratory tests, neuroimaging findings, treatment administered, and outcomes were collected. The strokes were classified according to TOAST based on clinical features and findings on either MRI brain with MR angiogram or CT brain with CT angiogram, ECG, echocardiogram, and 24-h Holter evaluation. The historical controls were selected from the stroke registry of one of the major tertiary coordinating stroke centers (St Johns National Academy of Health Sciences) in the same city, catering to the same population, during the same calendar period of the previous year (2019). All the historical controls had been evaluated in detail with either MRI brain with MR angiogram or CT brain with CT angiogram, carotid and vertebral Doppler, ECG, echocardiogram, and 24-h Holter. Descriptive statistics including mean, standard deviation, and percentages were used to summarize the data. A Pearson’s chi-squared test was performed for categorical variables, and an independence sample “t” test was performed for continuous variables for evaluation of the statistical difference between case and historical control groups. All tests were two-tailed, and a *P* value of .05 was considered statistically significant. All statistical analyses were conducted with the SPSS statistical package for Windows, Version 25 (IBM). Study was approved by the institutional review boards of the coordinating centers.

## Results

From 1st June 2020 to 31st August 2020, we had 62 COVID-19-related stroke patients, across 13 centers; 60 (97%) had ischemic strokes while 2 (3%) had hemorrhagic strokes. The mean age of patients was 55.66 ± 13.20 years, with 34 (77.4%) males; 90% (57/62) of the patients had one or more of the symptoms of fever, cough, fatigue, myalgia, and breathlessness preceding the stroke, for a period ranging from 1 day to 14 days (mean ± SD = 12.51 ± 5.06 days). SARS–CoV2 infection was confirmed in all patients using RT-PCR from the nasopharyngeal swab; 26% (16/62) of patients did not have any conventional risk factors for stroke. Hypertension was present in 61.3%; diabetes mellitus was seen in 54.8%, coronary artery disease in 8%, and atrial fibrillation in 4.8%. Baseline NIHSS score was 12.7 ± 6.44. Stroke severity was moderate (NIHSS 5–15) in 27 (61.3%) patients, moderate to severe (NIHSS 16–20) in 13 (20.9%) patients and severe (NIHSS 21–42) in 11(17.7%) patients. d-dimer was elevated in 91%; C-reactive protein was increased in 86.5% and serum ferritin in 72%. Diagnosis of stroke was confirmed by MRI brain in 68.5% of patients and with CT brain in 31.5% of patients. MR angiogram was available in 50%, CT angiogram in 25%, and carotid and vertebral Doppler in 60%. ECG was done in all patients. Echocardiogram was performed in 70% of patients, while 24-h Holter was done in only 40% of patients, because of lack of staff due to COVID infection and isolation precautions. In 60 patients with ischemic stroke, anterior circulation strokes were seen in 96.7%, while posterior circulation strokes were seen in 3.3%. According to TOAST classification, 48.3% had stroke of undetermined etiology, 36.6% had large artery strokes, 10% had small vessel occlusion, and 5% had cardioembolic strokes. In the anterior circulation strokes, 72% had middle cerebral artery territory infarcts, with 54% having right sided involvement, while remaining had both middle and anterior cerebral artery territory involvement. Three (5%) received intravenous thrombolysis with tenecteplase 0.2 mg/kg and 3 (5%) underwent mechanical thrombectomy, two endovascular and one surgical. Duration of hospital stay was 16.16 ± 6.39 days; 21% (13/62) died, while 37 (59.7%) had a mRS of 3–5 and 12 (19.4%) had a mRS 0–2 at discharge.

A comparison was done with the profile of 111 stroke patients admitted during the similar months (June–August 2019) in the non-COVID period, from our stroke registry (Table 1). Mean age and gender distribution were similar in stroke patients during the COVID and non-COVID periods. Among 111 controls, 104 (93.7%) had ischemic strokes and 7 (6.3%) had hemorrhagic strokes, which was comparable to the COVID-19-related strokes cohort. Time to presentation after stroke was earlier in patients with COVID-19-related stroke (*p* < 0.01). Baseline NIHSS score and mRS score at admission were worse in COVID-19-related stroke patients (*p* < 0.001). Anterior circulation strokes were more common in the COVID-19-related stroke group (*p* < 0.001), while posterior circulation strokes were more in the control group (*p* < 0.001). Comparison of TOAST subtypes showed a higher proportion of stroke of undetermined etiology (*p* < 0.001) in the COVID-19-related stroke group. Large artery and cardioembolic strokes were similar in both groups. Small artery strokes (*p* < 0.062) and strokes of other determined etiology (*p* < 0.03) were more common in the control group. Moderate (NIHSS 5–15), moderate to severe (NIHSS 16–20), and severe (NIHSS 21–42) strokes were more common in the COVID-19-related strokes group, while minor strokes (NIHSS 1–4) were more common in the non-COVID stroke group. Treatment administered with respect to stroke management was similar in both groups with respect to intravenous thrombolysis and mechanical thrombectomy. Mortality rate was significantly higher in the COVID-19-related stroke group (*p* < 0.001). NIHSS and mRS score at discharge were significantly worse in the COVID-19-related stroke group (*p* < 0.001).

**Table 1.** Demographic, clinical features, treatments, and outcomes in COVID versus non-COVID strokes.

Variable	COVID 19 positive cases (*n* = 62)	COVID 19 negative control 2019 (*n* = 111)	*P* value
Mean age in years (SD)	55.66 ± 13.20	57.21 ± 11.97	0.643
Male gender	48 (77.41)	79 (71.17)	0.372
Risk factors			
NIL	16 (25.80)	5 (4.50)	0.001
Hypertension	38 (61.29)	79 (71.17)	0.183
Diabetes	34 (54.83)	61 (54.95)	0.988
Coronary artery disease	5 (8.06)	10 (9)	0.832
Atrial fibrillation	3 (4.83)	1 (0.90)	0.09
Stroke types			
Ischemic	60 (96.77)	104 (93.69)	0.69
Hemorrhagic	2 (3.22)	7 (6.30)	0.382
Time to presentation–hr.	50.5 ± 43.58	124.76 ± 247.05	0.02
COVID-19 symptom onset to stroke symptom onset	12.51 ± 5.06	NA	
Baseline NIHSS	12.72580645 ± 6.44	5.189 ± 4.22	0.001
mRS at admission	4.06 ± 1.55	3.01 ± 1.26	0.001
Stroke severity			
Minor stroke (1–4)	NIL	60 (54.05)	0.001
Moderate stroke (5–15)	38 (61.29)	45 (40.54)	0.009
Moderate to severe stroke (16–20)	13 (20.96)	5 (4.50)	0.001
Severe stroke (21–42)	11 (17.74)	1 (0.90)	0.001
Stroke subtype (TOAST)	*N* = 60	*N* = 104	
Undetermined	29 (48.3)	23 (22.1)	0.001
Large artery	22 (36.6)	40 (38.4)	0.942
Small artery	6 (10)	23 (22.1)	0.062
Cardio embolism	3 (5)	10 (9.6)	0.318
Other determined	NIL	8 (7.6)	0.030
Stroke arterial territory	*N* = 60	*N* = 104	
Anterior circulation	58 (96.7)	70 (67.3)	0.001
Posterior circulation	2 (3.3)	29 (27.9)	0.001
Combined	NIL	5 (4.8)	0.001
COVID-19 symptoms (fever, cough, fatigue, dyspnea)	57 (91.9)	6 (5.40)	0.001
Treatment			
Conservative	54 (87.09)	103 (92.79)	0.215
IV thrombolysis	3 (4.83)	6 (5.40)	0.872
Mechanical thrombectomy	2 (3.22)	2 (1.80)	0.55
Surgical thrombectomy	1 (1.61)	NIL	0.18
External ventricular drain	1 (1.61)	NIL	0.18
Decompressive craniotomy	1 (1.61)	NIL	0.18
In hospital death	13 (20.96)	NIL	0.001
Duration of hospital stay	16.16 ± 6.39	8.36 ± 5.66	0.001
NIHSS at discharge	9.5 ± 8.87	2.31 ± 2.86	0.001
mRS at discharge	3.58 ± 1.23	1.86 ± 1.52	0.001

NIHSS: National Institutes of Health Stroke Scale; mRS: modified Rankin score.

Values are written as number (%) or mean ± standard deviation.

A subgroup analysis was done to find various factors associated with mortality in COVID-19-related stroke patients. A comparison of those who survived with those expired is summarized in Table 2. Hypertension (*p* < 0.05) and atrial fibrillation (*p* < 0.04) were more common in the expired group when compared to those who survived. Diabetes was more common in expired group, but it was not statistically significant (*p* < 0.07). Baseline NIHSS score was worse in those who died (*p* < 0.002) and most of the patients in the expired group had severe strokes (NIHSS 21–42, *p* < 0.001).

**Table 2.** Comparison of variables between expired and survived in the COVID-19 related stroke group.

Variable	Expired = 13	Survived = 49	*P* value
Mean age in years (SD)	55 ± 20.66	54.57 ± 13.12	0.210
Male gender	10 (76.92)	38 (77.55)	0.962
Risk factors			
No risk factors	1 (7.69)	15 (30.61)	0.093
Hypertension	11 (84.61)	27 (55.10)	0.052
Diabetes	10 (76.92)	24 (48.97)	0.072
Coronary artery disease	2 (15.38)	3 (6.12)	0.276
Atrial fibrillation	2 (15.38)	1 (2.04)	0.046
Stroke types			
Ischemic	13 (100)	47 (95.91)	
Hemorrhagic	NIL	2 (4.08)	
Time to presentation–hr.	53.92 ± 59.95	49.59 ± 38.87	0.753
COVID-19 symptom onset to stroke symptom onset	11.53 ± 6.10	12.77 ± 4.79	0.230
Baseline NIHSS	17.61 ± 6.80	11.42 ± 5.74	0.002
Stroke severity			
Minor stroke	NIL	NIL	
Moderate stroke	5 (38.46)	33 (67.34)	0.057
Moderate to severe stroke	1 (7.69)	12 (24.48)	0.186
Severe stroke	7 (53.54)	4 (8.16)	0.001
Stroke subtype (TOAST)			
Undetermined	6 (46.15)	23 (46.93)	0.960
Large artery	6 (46.15)	16 (32.65)	0.366
Small artery	NIL	6 (12.24)	0.184
Cardio embolism	1 (7.69)	2 (4.08)	0.596
Other determined	NIL	NIL	
Treatment			
Conservative	12 (92.30)	42 (85.71)	0.528
IV thrombolysis	1 (7.69)	2 (4.08)	0.590
Mechanical thrombectomy	NIL	2 (4.08)	0.459
Surgical thrombectomy	NIL	1 (2.04)	0.604
External ventricular drain	NIL	1 (2.04)	0.604
Decompressive craniotomy	NIL	1 (2.04)	0.604
Duration of hospital stay	16.30 ± 6.56	16.12 ± 6.42	0.927

NIHSS: National Institutes of Health Stroke Scale.

Values are written as number (%) or mean ± standard deviation.

## Discussion

SARS-CoV-2-related strokes are increasingly being recognized by the physicians all over the world. Incidence of stroke in major case series ranged from 1 to 6%.^1^ A recent cross-sectional comparative study from New York identified a 7.5-fold higher rate of ischemic stroke in COVID-19 compared to influenza.^2^ COVID-19-related strokes are more ischemic than hemorrhagic in all the studies reported.^3–5^ The current study is the largest from the Indian subcontinent. Previous studies suggested strokes were more common in elderly patients with severe disease, especially those with elevated levels of C-reactive protein, d-dimer, urea, and interleukin-6 (IL-6).^4,5^ But in the current study, the comparison with stroke in the non-COVID period showed that there was no difference in the age group of COVID-19-related stroke patients. Most studies, including the current study found a male preponderance,^5–7^ a pattern that was similar during COVID and non-COVID periods, with no statistical difference. 26% of COVID-19-related stroke patients had no conventional risk factors and this difference was significant when compared to non-COVID strokes. This pattern was observed in other studies and indicates a causative role of SARS–CoV-2 infection in these strokes.^8,9^ Hypertension and atrial fibrillation were more common in the COVID-19-related stroke group when compared to historical controls. Time to presentation to the hospital after the stroke was earlier in patients with COVID-19-related strokes when compared to historical controls. This was an interesting observation and would have been due to the less traffic on the roads due to the quarantine and the presence of systemic symptoms which might have brought these patients earlier to the hospital. Baseline NIHSS score and mRS score at admission were worse in patients with COVID-19-related strokes, suggesting strokes were of more severe nature, similar in line with the findings of other studies.^8,9^ Minor strokes were less in COVID-19-related stroke group when compared to the non-COVID period. This may be due to the fact that patients with mild symptoms might not have come to the hospital for evaluation due to the fear of the pandemic.^9^ Comparison of TOAST subtypes showed a higher proportion of strokes of undetermined etiology as observed in the previous studies.^8,9^ This was due to the incomplete stroke workup and disruption of usual stroke evaluation pathways, as many of these patients were in COVID isolation wards and COVID intensive care units. Though most case series pointed to a higher prevalence of large artery strokes, in the current study we did not find an increase in the occurrence of large artery strokes when compared to the historical control group. Anterior circulation strokes were more common in the COVID-19-related stroke group. The exact reason for this observation is not certain but may be due to the fact that more seriously ill patients with middle cerebral artery and internal carotid artery territory strokes would have reached the hospital for acute care. Minor anterior and posterior circulation strokes would not have presented to the hospital due to the quarantine protocols. Thrombolysis and mechanical thrombectomy rates were similar in both groups. Consistent with previous studies, we also found that mortality rates were significantly higher in the COVID-19-related stroke group (21%). Most of the published studies have reported a higher mortality ranging from 27.6% to 54.5%.^3,8,9^ On analysis of the various factors associated with mortality, it was found that presence of hypertension, atrial fibrillation, and baseline higher NIHSS scores were more common in the expired group in comparison to those who survived. Although diabetes was more common in the expired group, the difference was not statistically significant. In those who survived, NIHSS and mRS score at discharge were significantly worse compared to the historical cohort, suggesting that COVID-19-related strokes resulted in more disability. In a recent large multicenter study, 51% of patients had severe disability at discharge.^9^ Duration of hospital stay was much longer in COVID-19-related stroke group due to the associated systemic involvement and comorbidities.

Several theories have been proposed to explain the vascular complications of SARS–CoV-2 infection. SARS–CoV-2 is not only a neuroinvasive, neurotropic, neurovirulent virus but also has tropism to endothelial cells and cardiomyocytes.^10^ Cerebral and cardiovascular events have been attributed to direct viral invasion and thrombo-inflammation or immune thrombosis. A direct endotheliopathy resulting from invasion of the virus through the ACE-2 receptors on the surface of endothelial cells contribute to the endothelial dysfunction and thrombosis.^8^ Thrombo-inflammation is a consequence of the activation of various cells involved in immune defense by the virus and amplification of complement cascade and cytokine systems, resulting in further downstream stimulation of procoagulant pathways.^11^ There is also a simultaneous depletion of antithrombotic factors like protein C, S, plasminogen activator inhibitor 1, and antithrombin III. The elevated CRP and IL-6 levels are indirect indicators of the severe inflammatory response, while the elevated d-dimer levels indicate activation of the coagulation factors.^12^ This thrombo-inflammatory response with endothelial dysfunction is probably responsible for the strokes associated with COVID-19 infection.

## Limitations and strengths

The main limitations of the current study are its retrospective nature, possible selection bias, chance that less severe strokes, and COVID-19-related strokes with false-negative COVID tests would have been missed, lack of a concurrent prospective non-COVID stroke controls and incomplete stroke work up of COVID-19-related strokes patients due to isolation precautions and shortage of staff. However, the strengths of the current study are that it is one of the largest multicenter COVID-related stroke cohort from the Indian subcontinent, with a comparison with historical controls and assessment of risk factors associated with mortality.

## Conclusion

As we are witnessing an avalanche of the deadly pandemic, there is a definite increase in the number of COVID-19-related strokes, in developing countries, like India. Stroke of undetermined etiology appears to be the most common TOAST subtype in COVID-19-related stroke patients. COVID-19-related strokes are of more severe nature and results in higher mortality and morbidity. Hypertension, atrial fibrillation, and higher baseline NIHSS scores are associated with increased mortality. The entire spectrum of COVID-19-related strokes will become clearer with more data accumulating from various centers across the globe. Meanwhile the stroke teams all over the world, especially those in developing countries should be more than prepared to manage these COVID-19-related strokes.
